# Monitoring HIV Prevention Programme Outcomes among Key Populations in Kenya: Findings from a National Survey

**DOI:** 10.1371/journal.pone.0137007

**Published:** 2015-08-27

**Authors:** Parinita Bhattacharjee, Leigh M. McClarty, Helgar Musyoki, John Anthony, Japheth Kioko, Shem Kaosa, Bernard E. Ogwang, George Githuka, Martin Sirengo, Sarah Birir, James F. Blanchard, Nicholas Muraguri, Shajy Isac, Stephen Moses

**Affiliations:** 1 Centre for Global Public Health, Department of Community Health Sciences, University of Manitoba, Winnipeg, Manitoba, Canada; 2 National AIDS and STI Control Programme, Ministry of Health, Government of Kenya, Nairobi, Kenya; 3 Partners for Health and Development in Africa, Nairobi, Kenya; 4 Ministry of Health, Government of Kenya, Nairobi, Kenya; University of Missouri-Kansas City, UNITED STATES

## Abstract

In preparation for the implementation of the Kenya AIDS Strategic Framework 2014/15-2018/19, the Kenya National AIDS and STI Control Programme facilitated a national polling booth survey as part of a baseline assessment of HIV-related risk behaviours among FSWs, MSM, and PWID, and their utilization of existing preventive interventions, as well as structural factors that may influence KPs’ vulnerability to HIV. The survey was conducted among “key populations” (female sex workers, men who have sex with men, and people who inject drugs) to understand current HIV risk and prevention behaviours, utilization of existing programmes and services, and experiences of violence. In total, 3,448 female sex workers, 1,308 men who have sex with men, and 690 people who inject drugs were randomly selected to participate in polling booth survey sessions from seven priority sites. Survey responses were aggregated and descriptive statistics derived. In general, reported condom use among all key populations was quite high with paying clients, and lower with regular, non-paying partners. Many participants reported unavailability of condoms or clean injecting equipment within the past month. Exposure to, and utilization of, existing HIV prevention services varied significantly among the groups, and was reported least commonly by female sex workers. Encouragingly, approximately three-quarters of all key population members reported receiving an HIV test in the past three months. All key population groups reported experiencing high levels of physical and sexual violence from partners/clients, and/or arrest and violence by law enforcement officials. Although some of the findings are encouraging, there is room for improvement in HIV prevention programmes and services for key populations across Kenya.

## Introduction

Kenya’s HIV epidemic is characterized as “generalized” among the adult population (15–49 years), with a reported average HIV prevalence of 6.0% in 2013 [1]. However, both HIV prevalence and incidence are concentrated among “key populations” (KPs), which are considered to be at a heightened and disproportionate risk of HIV acquisition and transmission, due in part to sexual and social behaviours [2]. In Kenya, identified KPs include female sex workers (FSWs), men who have sex with men (MSM), including male sex workers (MSWs), and people who inject drugs (PWID) [3]. In Nairobi, average the HIV prevalence among MSM, FSWs, and PWID is estimated to be 18.2%, 29.3%, and 18.7%, respectively [4], while HIV prevalence among MSWs ranges from 26.4% to 40.0% in Nairobi, and 19.7% in Mombasa [5]. A rapid situational analysis of injection drug use in Nairobi and Coast provinces also identified prevalence estimates among PWID of 17% and 47% among males and females, respectively [6]. Furthermore, recent mapping estimates identified 133,675 urban FSWs throughout the country, with significant regional variation, ranging from over 29,000 FSWs in Nairobi Province to just over 2,000 in North Eastern Province [7], while over 19,000 Kenyans self-identified as MSM, and over 18,000 individuals identified as PWID [8]. These numbers clearly highlight the importance of developing strategic HIV prevention programmes that are tailored to reach KPs and priority geographic regions to address the country’s highly heterogeneous epidemic [3].

The Government of Kenya, in collaboration with national and international partners, has committed significant resources towards developing, implementing, and scaling up HIV prevention programmes, with specific focus on KPs, by employing a combination HIV prevention approach [3,9]. In 2009, the United Nations Joint Programme on HIV/AIDS (UNAIDS) formally recommended combination prevention strategies to achieve the greatest and longest-lasting impact on reducing HIV incidence [10]. Following UNAIDS recommendations, Kenya’s National AIDS Control Council (NACC) and the National AIDS and STI Control Programme (NASCOP) currently oversee 82 combination intervention programmes across the country. The Kenya AIDS Strategic Framework (KASF) 2014/15-2018/19 [3] outlines the national strategy developed to address the drivers of Kenya’s HIV epidemic, and builds upon the efforts and accomplishments of previous national strategic plans, including the Kenya National AIDS Strategic Plan (KNASP) III. One strategic direction outlined in KNASP III and KASF is the continued monitoring and evaluation of current combination prevention programmes implemented across the country. Accordingly, a survey was conducted under KNASP III in 2013–14 to act as a baseline for monitoring HIV-related risk behaviours among FSWs, MSM, and PWID, and their utilization of existing preventive interventions, as well as structural factors that may influence KPs’ vulnerability to HIV. Similar surveys are planned every 12–15 months post-baseline to monitor progress and make midcourse corrections during the implementation of the KASF.

The present paper reports on findings from KASF’s baseline evaluation conducted among KPs who utilise services offered through combination HIV prevention programmes in seven districts of Kenya. Additionally, we compare findings between KPs and across the seven study sites in order to gain an understanding of heterogeneity across the country.

## Methodology

### Study design and sample selection

NASCOP has prioritized seven study sites across Kenya (Nairobi, Mombasa, Nakuru, Nyeri, Thika, Kisumu, and Eldoret) to account for regional heterogeneity in HIV prevalence HIV risk. A two-stage, stratified cluster sampling procedure was used to recruit PBS participants. For the first stage of sampling in each study site, a list of all active, previously mapped “hotspots” formed the sampling frame for selecting primary sampling units (PSUs). A hotspot was defined as any physical location where KP members meet partners or clients (soliciting, cruising), engage in sexual intercourse with partners/clients, or obtain or inject drugs. The lists of hotspots for each study site were then stratified by geographic location and typology (i.e. FSW, MSM, or PWID). Once stratified, all hotspots of the same typology in a particular geographic location formed the PSU within a study site. Of note, hotspots where KP estimates were small were merged with nearby hotspots of the same typology so that an adequate number of KPs would be included within each PSU. PSUs were selected through systematic random selection from the stratified list of hotspots. The number of PSUs per study site was determined based upon the required sample size and estimated KP size per hotspot (which was used as a proxy for the total population size). Target sample sizes for FSWs, MSM, and PWID in each study site were adjusted with finite population correction (FPC), accounting for the estimated size of KPs in each site. Sample sizes were calculated to detect a 15% change in the indicators of interest between the baseline and a planned follow-up survey (e.g. 15% increase in condom use with casual clients from a baseline of 50%), with 80% statistical power. Since the estimated size of each KP varied by site, and because the minimum required sample was adjusted with FPC, the target sample size for each KP varied across study sites. The target sample size for each KP across all study sites (accounting for a 15% non-response rate) was 3,313 FSWs, 1,495 MSM, and 696 PWID.

In the second stage of sampling, peer educators received thorough training on the sampling procedure and were asked to develop a list of individuals self-identifying with KP members who frequented previously identified sampling hotspots [7]. Peer educators used a systematic random sampling method [11,12] to identify potential participants who frequented hotspots, rather than focusing only on individuals who regularly accessed services provided by intervention programmes, as a way to reduce selection and participation biases. Potential participants were made aware that refusal to participate would not, under any circumstance, jeopardize their ability to utilize services provided by programmes. Individuals who agreed to participate were provided with information on the day, time, and venue for their PBS session, which was arranged in consultation with NGO/CBO implementing partners in each study site. Up to twelve participants were organized into homogenous groups based on sociodemographic and geographic characteristics. A total of 350 PBS group sessions were conducted over the duration of the study. All PBS sessions were led by two research assistants, one of whom self-identified as a KP member.

### Data collection and analysis

Reliable self-reported data on knowledge, behaviours and practices influencing HIV transmission and acquisition are critical for effective HIV prevention programme design and implementation, but are often difficult to obtain due to the sensitivity surrounding sexual behaviours [12–14]. Accurate reporting of sexual behaviour is heavily influenced by personal and contextual barriers, such as poor recall, perceptions of lack of confidentiality, and social desirability bias [13–16]. Survey methods that offer a greater level of privacy for respondents and assure anonymity of their response are likely to elicit more accurate data [15]. Polling booth surveys (PBS) are one such strategy that seeks to overcome reporting biases associated with face-to-face interviews and self-administered questionnaires [11,12]. PBS is a group interview method in which participants are provided a private booth containing colour-coded ‘yes’, ‘no’, and ‘not applicable’ ballot boxes, and a set of numbered ‘voting’ tokens corresponding to each questionnaire item [12]. Participants then answer survey questions, which are read aloud, by placing the appropriately numbered token in the relevant ballot box. A more detailed description of this process is described elsewhere [12]. The anonymity afforded to PBS participants has been found to elicit more accurate reporting of sensitive and personal information regarding sexual behaviours when compared to both face-to-face interviews and self-administered questionnaires [11–13].

Three different questionnaires were used for PBS sessions with FSWs, MSM, and PWID. As a baseline survey, each questionnaire was designed to elicit information on indicators that related to the components of combination prevention packages, allowing for the measurement of the effect of behavioural, biomedical, and structural interventions over time. The final surveys were translated from English to Kiswahili, Kenya’s official language, then back-translated to English to ensure accuracy and consistency. A pilot study was conducted to field-test the instrument, and adjustments were made based on feedback prior to distribution to field staff.

Data was entered using Microsoft Access and exported to SPSS (v20) and Microsoft Excel for analysis. Descriptive analysis was performed to produce frequencies, proportions, and comparative statistics. Given that PBS data are anonymised and aggregated at the group level (i.e. responses are summarised for each PBS session), the data are unlinked, and do now allow for more complex, multivariate analyses. Data were weighted using KP size estimates and aggregated to provide site-wide estimates for specific indicators, with town and site typology breakdowns.

### Ethical considerations

Kenya’s Ministry of Health approved the PBS as a method for routinely collecting data to inform programme monitoring and evaluation. Participation in PBS sessions was voluntary. Women and men, at least 18 years of age, who identified with KPs, were eligible to participate in the survey, after providing written or verbal informed consent. Data were anonymous and unlinked, thus minimizing potential risks associated with participation. No incentives were provided to participants for taking part in the surveys, although return costs of travelling to the PBS session sites were reimbursed.

## Results

### Sociodemographic characteristics and reported risk behaviours

In total, 5,446 participants took part in the PBS: 204 sessions with FSWs (*n* = 3,448, 63.3%); 70 sessions with MSM (*n* = 1,308, 24.0%); and 63 sessions with PWID (*n* = 690, 12.7%). The number of PBS sessions held with each KP was based on availability of members of KPs and on estimates of KP sizes (Table 1). Sociodemographic characteristics and self-reported risk behaviours of all PBS participants are outlined in Table 2. Briefly, over half of MSM participants had exchanged sex with other men for money or other goods in the past three months, but the variation in this parameter across study sites was significant (*p*<0.001). Slightly less than one-fifth of PWID participants reported at least one occasion in the past month in which they had sex with a paying client. Data on type of drug used by PWID was not collected. In addition to many participants reporting transactional sexual relationships, the majority of FSWs (60.4%), MSM (64.0%), and PWID (69.5%) also reported having a regular, non-paying intimate partner.

**Table 1:** Polling booth survey sample coverage of key populations in each study site across Kenya.

	Number of participants reached		
Study site	*FSWs*	*MSM*	*PWID*
**Nairobi**	824	301	380
**Mombasa**	693	261	310
**Nakuru**	456	212	-
**Thika**	456	238	-
**Kisumu**	405	296	-
**Nyeri**	264	-	-
**Eldoret**	350	-	-
**Total**	**3,448**	**1,308**	**690**

**Table 2:** Sociodemographic characteristics and self-reported HIV risk behaviours of polling booth survey participants.

	Key populations							
	FSW		MSM		PWID		Total	
	*n*	(%)	*n*	(%)	*n*	(%)	*N*	(%)
**Participants**	3,448	(63.3)	1,308	(24.0)	690	(12.7)	5,446	(100)
**PBS sessions**	204	(60.5)	70	(20.8)	63	(18.7)	337	(100)
**Age range (years)**								
18–25	1,495	(43.4)	855	(65.4)	112	(16.2)	2,462	(45.2)
26–40	1,839	(53.3)	425	(32.5)	485	(70.3)	2,749	(50.5)
>40	114	(3.3)	28	(2.1)	92	(13.3)	234	(4.3)
**Sex**								
Male	0	(0)	1,308	(100)	608	(88.1)	1,916	(35.2)
Female	3,448	(100)	0	(0)	82	(11.9)	3,530	(64.8)
**Anal sex**								
Ever	548	(15.9)	1,219	(93.2)	-	-	1,767	(37.2)
Past one-month	272	(7.9)	1,003	(76.7)	38	(5.5)	1,313	(24.1)
**Exchanged money/goods for sex**								
With other men in the past three months	-	-	770	(58.9)	-	-	770	(58.9)
With any paying client in the past month	-	-	-	-	246	(18.8)	246	(18.8)
**Regular, non-paying intimate partnerships**	2,207	(64.0)	837	(64.0)	480	(69.6)	3,524	(64.7)
**Injection drug use**								
Heroin/narcotic in the past month	-	-	-	-	635	(92.0)	635	(92.0)

### Behavioural outcomes

A number of behavioural outcomes were explored throughout PBS sessions with KPs, all of which are outlined in Table 3.

**Table 3:** Self-reported HIV risk or preventive behaviours among polling booth survey participants.

	Key populations (%)			Variation between key populations			Variation within key populations across study sites		
	FSWs (*n* = 3,448)	MSM (*n* = 1,308)	PWID (*n* = 690)	FSWs vs. MSM	FSWs vs. PWID	MSM vs. PWID	FSWs	MSM	PWID
**Condom use**									
At last sex with any paying client/at last paid sex	87.6	57.6	66.9	*	*	NS	*	*	NS
At last sex with a regular, non-paying partner	57.3	69.6	43.3	*	*	*	*	*	NS
At last anal sex	58.1	76.7	27.3	*	*	*	*	*	†
**Condom non-use**									
Partner did not want to wear condom in the past month	31.5	23.3	-	*	-	-	*	*	NS
Condom not available at time of sex in the past month	23.1	32.5	35.2	*	*	NS	*	*	NS
**Condom breakage and slippage**									
At last sex with any partner	28.1	20.2	22.6	*	†	*	*	*	NS
**HIV knowledge**									
HIV can be transmitted by mosquitoes	13.6	12.5	-	NS	-	-	*	*	NS
HIV can be transmitted through touching/hugging	4.8	6.7	-	NS	-	-	*	*	NS
Can tell if someone has HIV by their physical appearance	18.6	14.1	-	†	-	-	*	NS	-
HIV can be transmitted through anal sex	81.2	90.3	85.9	*	NS	†	*	NS	NS
Condoms can prevent HIV transmission	80.7	94.5	-	*	-	-	*	*	NS
**Needle and syringe usage**									
Used new needle and syringe when injecting drugs last time	-	-	87.8	-	-	-	-	-	*
Shared needle at last injection	-	-	17.1	-	-	-	*	*	†
Clean needle not available in the past month	-	-	36.4	-	-	-	*	NS	NS

* *p*<0.001

† *p*<0.05

NS = not significant

#### Condom and lubricant use

A greater proportion of FSWs (87.6%) reported using a condom at last sex when they had sex with a paying client when compared to MSM (57.6%) and PWID (66.9%), and these differences were significant (*p*<0.001). There was also significant variation in reported condom use with paying clients among FSWs and MSM across study sites. Condom use with regular, non-paying partners was lower than with paying partners across all KPs, but with MSM reporting significantly higher rates of condom use with regular partners compared to both FSWs and PWID. Overall, a significantly higher proportion of MSM (76.7%) reported condom use at last anal sex act with any partner compared to FSWs (58.1%) and PWID (27.3%).

#### Condom non-use

Nearly one-third of FSWs and almost one-quarter of MSM reported an occasion in the past month when they wanted to use condoms during sex, but their sex partner refused. There was significant variation in condom non-use among MSM and FSW participants across all study sites (*p*<0.001). Additionally, approximately one-quarter of FSWs, and one-third of both MSM and PWID reported an occasion in the past month in which condoms were not available during a sexual encounter.

#### Condom breakage and slippage

Condom breakage and slippage was commonly reported among PBS participants from all KPs. Over one-quarter of FSWs (28.1%) reported condom breakage or slippage at last sex, compared to 20.2% of MSM and 22.6% of PWID, and significant differences were found between FSWs and MSM (*p*<0.001), FSWs and PWID (*p*<0.003), and MSM and PWID (*p*<0.001), and across study sites among FSWs.

#### Knowledge about HIV

The majority of PBS participants knew that HIV is spread through anal sex, but the differences in reports between FSWs and MSM (*p*<0.001) and MSM and PWID (*p*<0.008) were significant. Unsurprisingly, 94.5% of MSM, compared to 81.2% of FSWs, were aware that using condoms protects against HIV. Not all knowledge-related questions were posed to PWID, as indicated in Table 3.

#### Needle sharing

Nearly 90% of PWID reported using a clean needle and syringe during their most recent injection, but there was significant variation across sites (*p*<0.005). However, one-fifth of PWID participants also reported sharing needles with another person during their last injection. Over one-third of PWID also reported that there had been at least one occasion in the past one-month when clean needles were not available at the time of injection, with significant variation across sites (*p*<0.001).

### Exposure to prevention and treatment programmes and services

In Table 4, we report on a number of indicators associated with exposure to HIV prevention and treatment programmes and services for KPs in Kenya.

**Table 4:** Reported exposure to and utilization of biomedical interventions among polling booth survey participants.

	Key populations (%)			Variation between key populations			Variation within key populations across study sites		
	FSWs (*n* = 3,448)	MSM (*n* = 1,308)	PWID (*n* = 690)	FSWs vs. MSM	FSWs vs. PWID	MSM vs. PWID	FSWs	MSM	PWID
**Exposure to intervention**									
Visited programme clinic/DIC in the past three months	53.5	63.8	71.4	*	*	*	*	NS	*
**HIV test**									
Ever	93.9	91.8	93.8	NS	NS	NS	*	†	NS
Past three months	72.4	73.7	71.0	NS	NS	NS	*	*	NS
**HIV care and treatment**									
Registered in ART programme	31.8	27.4	41.6	†	*	NS	*	*	NS
On ART (% of those registered)	58.4	48.5	38.5	*	*	NS	*	*	*
**STI symptoms and treatment**									
Currently experiencing STI symptoms	23.1	17.0	15.1	*	*	NS	*	*	NS
Treated for STI in past three months	22.1	45.8	41.1	*	*	NS	*	†	NS

* *p*<0.001

† *p*<0.05

NS = not significant

More PWID reported visiting a programme clinic or drop-in clinic (DIC) in the past three months compared to FSWs and MSM (*p*<0.001), and variation in reported use was observed across all study sites among all KPs.

Nearly all FSWs, MSM, and PWID reported having ever been tested for HIV, and no significant differences in reported HIV testing rates were observed between groups. Similarly, high proportions of all KPs reported being tested for HIV in the past three months, although there was significant variation in the proportion of FSWs and MSM reporting testing in the past three months across study sites (*p*<0.001).

Slightly less than one-half of PWID reported being registered in an ART programme, which was significantly higher than reports from both FSWs and MSM, and among those registered, 58.4% of FSWs, 48.5% of MSM and 38.5% of PWID reported that they were currently taking antiretroviral (ARV) drugs. The variation in reportedly taking ARV drugs among all KP groups was significant across study sites (*p*<0.001).

Nearly one-quarter of FSWs reported currently experiencing symptoms associated with STIs, which was significantly higher than the proportion of MSM (17.0%) and PWID (15.1%). However, a significantly lower proportion of FSWs reported receiving STI treatment in the past three months when compared to both MSM and PWID.

### Structural factors

Finally, participants were asked questions about their personal experiences of violence (Table 5). Almost one-quarter of FSWs reported experiences of being beaten or physically forced to have sexual intercourse in the past six months, while 16.7% of MSM and 7.7% of PWID reported the same. Differences in the reporting of physical and sexual violence across all KPs were statistically significant (*p*<0.001). Significant variation was also observed across sites for FSWs and MSM. Female PWID were significantly more likely than male PWID to report physical and sexual violence in the past six months (*p*<0.001, data not shown).

**Table 5:** Reported experiences of violence among polling booth survey participants.

	Key populations (%)			Variation between key populations			Variation within key populations across study sites		
	FSWs (*n* = 3,448)	MSM (*n* = 1,308)	PWID (*n* = 690)	FSWs vs. MSM	FSWs vs. PWID	MSM vs. PWID	FSWs	MSM	PWID
**Physical and sexual violence**									
Past six months	22.4	16.7	7.7	*	*	*	*	*	NS
**Violence within work/living environment**									
Arrested or beaten up by police, criminal elements, etc. in the past six months	43.8	24.0	57.4	*	*	*	*	†	*

* *p*<0.001

† *p*<0.05

NS = not significant

A substantial proportion of participants reported experiencing violence at the hands of police, private security officers, and criminal elements. Over one-half of PWID, 43.8% of FSWs and 24.0% of MSM reported such violence, and differences across all groups were significant (*p*<0.001). Regional variation was also observed among reports from FSWs and PWID participants. Among PWID, experiences of violence from these parties were reported significantly more commonly by males than by females (*p*<0.001, data not shown).

## Discussion

This is the first national behavioural survey that has been conducted among KPs in Kenya with the purpose of generating baseline data that can be used for programme monitoring [17,18]. Many of our findings were encouraging, and suggestive that the majority of FSWs, MSM, and PWID in Kenya are aware of basic HIV prevention strategies, including the importance of regular HIV testing, consistent condom use with clients, as well as with regular, non-paying partners, and of using clean injection equipment. Additionally, existing combination HIV prevention programmes targeting KPs in each of the study sites appear to be successful in mobilizing communities to accept HIV testing and counselling services, as reported uptake of these services was quite high.

However, there are several points of concern. Between one-quarter and two-fifths of participants reported unavailability of condoms or clean needles/syringes, which indicates that the availability and accessibility of these commodities need to be improved. Further investigation into barriers that limit individuals’ ability to obtain the fundamental tools needed for HIV prevention and for harm reduction is warranted. HIV prevention programmes must ensure that services for STI treatment, HIV testing are accessible and acceptable for KPs, so that, higher proportions of KP members access these services. Previous research in Kenya has indicated that the use of community health workers can substantially increase utilization of health services for maternal and newborn health [19] and HIV treatment [20], and this may also be a beneficial strategy to enhance national HIV prevention efforts.

The PBS methodology has several limitations. Due to the anonymous nature of PBS methodology, responses cannot be linked with participants’ sociodemographic characteristics. However, we have tried to address this issue by stratifying participants into sites and typologies. Furthermore, PBS only allows for binary responses, thus limiting the depth of information that can be obtained. Finally, we did not ask any questions on participants’ exposure to peer education, despite this being a key strategy in HIV programming with KPs. We intend to incorporate this element into the next round of PBS. In spite of its limitations, PBS is a rapid and inexpensive methodology that can yield reliable data that are straightforward to collect and analyze. As such, PBS are suitable for monitoring programme outcomes on a regular basis, and can guide the continuous refinement of existing HIV prevention programming as context and priorities change over time.

Unfortunately, very often the legal, political, and social environments in which KPs are situated do not allow for individuals to have control over circumstances that shape their vulnerability to HIV and STIs [21]. Relatively high reported rates of sexual and physical violence among FSWs and MSM coincided with large proportions of participants foregoing condom use due to partner opposition/refusal. Whether these two parameters are associated should be studied further among KPs in Kenya, as previous research has identified significant relationships between sexual violence and unprotected sex, specifically in the context of sex work [22,23]. Within existing combination prevention programmes, it will be necessary to strengthen structural interventions that aim to reduce violence towards KPs and mitigate unequal, and often gendered power dynamics [24,25]. This will to ensure that KPs have greater control over their ability to negotiate condom use, and that their work and home environments are safe and free of violence at the hands of partners, clients, and authority figures. Programmes that narrowly target individual-level HIV risk factors (e.g. focusing only on HIV education) have been shown to have limited impact on HIV incidence and transmission among KPs [10].

## Conclusion

As Kenya prepares to implement KASF 2014/15-2018/19, a robust monitoring and evaluation (M&E) system is needed to assess all components of existing combination HIV prevention programmes. It is critical that M&E strategies are practical, straightforward, and effective at collecting information that is current, relevant, and readily useable [18]. As HIV prevention programmes for KPs in Kenya scale up, it will be important to monitor them and to assess the scale of the response. PBS represent a practical and simple way assess the coverage, availability, and quality of existing HIV prevention programmes [18]. The information generated will be used as an initial point of reference to track future progress, and ultimately to gauge the impact that the combination HIV prevention programmes have on HIV-related risk and vulnerability.
